# Neonatal intensive care admission for term neonates and subsequent childhood mortality: a retrospective linkage study

**DOI:** 10.1186/s12916-023-02744-7

**Published:** 2023-02-06

**Authors:** Shahar Talisman, Joshua Guedalia, Rivka Farkash, Tehila Avitan, Naama Srebnik, Yair Kasirer, Michael S. Schimmel, Donia Ghanem, Ron Unger, Sorina Grisaru Granovsky

**Affiliations:** 1 Department of Obstetrics & Gynecology, Shaare Zedek Medical Center, Affiliated with the Hebrew University-Hadassah School of Medicine, Jerusalem, Israel; 2 The Mina and Everard Goodman Faculty of Life Sciences, Bar Ilan University, Ramat-Gan, Israel; 3 Department of Pediatrics, Shaare Zedek Medical Center, Affiliated with the Hebrew University-Hadassah School of Medicine, Jerusalem, Israel

**Keywords:** Neonatal intensive care unit (NICU), Term neonate, Long-term mortality, NICU admission diagnosis

## Abstract

**Background:**

Neonatal intensive care unit (NICU) admission among term neonates is a rare event. The aim of this study was to study the association of the NICU admission of term neonates on the risk of long-term childhood mortality.

**Methods:**

A single-center case–control retrospective study between 2005 and 2019, including all in-hospital ≥ 37 weeks’ gestation singleton live-born neonates. The center perinatal database was linked with the birth and death certificate registries of the Israeli Ministry of Internal Affairs. The primary aim of the study was to study the association between NICU admission and childhood mortality throughout a 15-year follow-up period.

**Results:**

During the study period, 206,509 births were registered; 192,527 (93.22%) term neonates were included in the study; 5292 (2.75%) were admitted to NICU. Throughout the follow-up period, the mortality risk for term neonates admitted to the NICU remained elevated; hazard ratio (HR), 19.72 [14.66, 26.53], (*p* < 0.001). For all term neonates, the mortality rate was 0.16% (*n* = 311); 47.9% (*n* = 149) of those had records of a NICU admission. The mortality rate by time points (ratio_1:10,0000_ births) related to the age at death during the follow-up period was as follows: 29, up to 7 days; 20, 7–28 days; 37, 28 days to 6 months; 21, 6 months to 1 year; 19, 1–2 years; 9, 2–3 years; 10, 3–4 years; and 27, 4 years and more. Following the exclusion of congenital malformations and chromosomal abnormalities, NICU admission remained the most significant risk factor associated with mortality of the study population, HRs, 364.4 [145.3; 913.3] for mortality in the first 7 days of life; 19.6 [12.1; 32.0] for mortality from 28 days through 6 months of life and remained markedly elevated after age 4 years; HR, 7.1 [3.0; 17.0]. The mortality risk related to the NICU admission event, adjusted for admission diagnoses remained significant; HR = 8.21 [5.43; 12.4].

**Conclusions:**

NICU admission for term neonates is a pondering event for the risk of long-term childhood mortality. This group of term neonates may benefit from focused health care.

**Supplementary Information:**

The online version contains supplementary material available at 10.1186/s12916-023-02744-7.

## Background

Neonatal intensive care unit (NICU) admission entails immediate risk for morbidity and mortality for the newborns and emotional burden for their families, at a high cost to the health care system. The majority of studies have focused primarily on admission and risk factors for preterm NICU admission and long-term follow-up. In a time trend analysis of 38 units in the USA between 2007 and 2012, the overall NICU admission rate increased by 23% after adjusting for maternal and neonatal characteristics, while the admitted neonates were increasingly likely to be at term [1]. Mortality and long-term morbidity are major concerns for infants at NICU, while death among term neonates during the NICU hospitalization is considered a common occurrence, and its reported incidence is 8–15% [2, 3].

To our knowledge, no previous studies have examined long-term childhood mortality for this group of neonates. Thus, the aim of this study was to study the association between the NICU admission of term neonates and the risk of long-term childhood mortality.

## Methods

### Study population and data collection

We conducted a retrospective cohort study using the computerized medical record database of a single large obstetric center between 2005 and 2019, with a mean of 13,500 deliveries per year. The study hypothesis (H1) was that the NICU admission for term neonates increases the risk for long-term mortality. For the study we included all singleton term live births (≥ 37 weeks’ gestation). The following were excluded from the study: multifetal birth, preterm birth (< 37 weeks’ gestation), and stillbirth (intra-uterine fetal death). Congenital malformations and genetic syndromes were recorded and study analyses performed with and without the inclusion of these diagnoses at the time of NICU admission. Data on demographic and obstetric characteristics as well as data on the course of delivery and delivery complications were derived from the electronic database management software, which is updated during labor and validated by the computer systems personnel periodically. The data file was de-identified by the hospital personnel and constructed for analysis.

Coverage for all women and neonates for antenatal, intrapartum, NICU care, and any health costs throughout life is provided under the National Insurance Health Plan.

The unique maternal and neonatal national identity numbers were used to link between the perinatal, laboratory, NICU, and the Israeli Ministry of Internal Affairs death certificate databases. Follow-up time for each neonate was from birth until August 2020.

The study protocol was submitted to the institutional Ethical Committee (Helsinki Committee) and was exempted on the basis of an anonymous analysis (reference number SZMC_0199-19); Identifying numbers were erased after the data were linked.

### Data collection and variables

Demographic and clinical maternal data included maternal age, education, population group, ethnicity, gravidity, parity, pregnancy complications, and maternal comorbidities (gestational/pre-gestational diabetes, hypertensive disorders, and other background maternal diseases). Current and past obstetrical data included parity, assisted reproductive techniques (ART) used to achieve pregnancy, prior cesarean section, prior miscarriage, high-risk hospitalization during pregnancy, inter-pregnancy interval, antenatal care, past neonatal death, gestational age at birth, admission vital signs (diastolic and systolic blood pressure, temperature [oral], heart rate), mode of labor initiation (spontaneous, induction, no trial of labor), mode of delivery (vaginal spontaneous, instrumental or cesarean section), trial of labor after cesarean (TOLAC), epidural analgesia, oxytocin induction/augmentation, thick meconium, head presentation, normal fetal monitor, modified Bishop score at admission [4], total labor duration (min), stage II duration (min), stage III duration (min), on-call hours at birth and severe placental, uterine and maternal complications (dehiscence, uterine rupture, placenta accrete, massive bleeding, post-partum hemorrhage, blood products, re-laparotomy and uterine atony). Neonatal characteristics included birth weight, sex, 1′- and 5′-Apgar score, macrosomia (birth weight > 4000 g), small for gestational age (SGA) by population growth curves [5], NICU admission (any transfer with a length of stay of at least 4 h), diagnoses at admission (one or more) as per ICD coding and diagnoses registered on the neonatal discharge records (three authors: RF, NS, SGG independently reviewed the diagnoses and ICD codes and reached agreement on disease categories; disagreements were discussed jointly and decided): genetic syndromes and chromosomal anomalies, respiratory, brain, cardiovascular, skeleton/skin, digestive, electrolytes, hypoglycemia/diabetes, jaundice, infection, meconium aspiration, other anomalies, and mortality.

The institutional policy for NICU admission of term neonates is based on the decision of the staff neonatology specialist and/or pediatrician assessment of neonatal well-being at any point in time after delivery (either immediately in the delivery room or in the neonatal ward until discharge, at least 36 h post-delivery), as well as departmental protocols for infants of diabetic mothers. For the purpose of the study, due to the long duration of the study and possible policy and facility variations that might determine the NICU admission decision-making process (versus observation at the neonatal maternity ward), we combined the direct delivery room-NICU admission and delayed maternity-NICU admission. Our institution’s special care nursery is included in the regular maternity service. If required the neonate is transferred to NICU; data on separate early/delivery rooms and delayed NICU admissions were culled and not reported here. The NICU admissions do not include any post-discharge (home) re-admissions; those are reared to the pediatric intensive care unit or the pediatric ward.

### Data analysis

An analysis for assessing neonatal mortality from birth to the end of the study period, up to 15 years, was performed. Univariate analysis was initially conducted; continuous variables were analyzed using a *T*-test or Mann–Whitney *U*-test, and categorical variables were analyzed using chi-square test or Fisher’s exact test. A backward stepwise multivariable Cox proportional hazards model was used to assess the association between NICU admission for term neonates and neonatal long-term mortality, adjusted for maternal and delivery characteristics that are known to be related to neonatal severe morbidity and mortality, as well as those found to be significant in the univariate analyses, as well as for congenital malformations and genetic syndromes (a complete list can be found at Additional file 1: Table S1). Characteristics that were significantly associated with mortality rates were reported (hazard ratio; HR; 95% Confidence interval). NICU vs. no-NICU mortality was examined by time-frames, using Chi-square tests. The Kaplan–Meier method was used to estimate the cumulative incidence of mortality over time in each study group (NICU and no-NICU) as well as for each population group (Jewish and Arab). NICU admission diagnoses were evaluated, and the mortality risk was estimated by NICU admission diagnosis and time elapsed from birth across the follow-up timeline. In order to dissect the association of the specific diagnoses for NICU admission on the long-term mortality risk, we fitted additional models for specific diagnoses, e.g., jaundice (alone and with other disorders) and for groups of diagnoses. We previously reviewed the charts and codes of diagnoses and prepared the classification for the study as described elsewhere [6]. Briefly, three authors: RF, NS, and SGG independently reviewed the diagnoses and ICD codes and reached an agreement on disease categories; disagreements were discussed jointly and decided. For the purpose of the study, the recorded diagnosis for the NICU hospitalization was grouped by potentially high severity disorders; chromosomal, respiratory, brain and central nervous system, cardiovascular, skeleton and skin, digestive tract, and potentially low (transient) severity disorders; electrolyte, hypoglycemia, jaundice, infection, meconium aspiration.

Mortality time spans age groups categories were based on the neonatal definitions in accord with the neonatologist team and in accordance to accepted definitions; early neonatal mortality (0–7 days), late neonatal mortality (7–28 days) (infant mortality is defined for the first 28 days of life and divided as early and late) [7]. In the first year of life, we decided to dissect the mortality risk for every 6 months since mortality is closely derived from neonatal events while after 2 years of life, we used 1 year span until the age of 4 years. After the age of 4 years, we assumed that mortality might also be related to non-health causes, e.g., car accidents and abuse, and since we did not have the cause of death available and the number of death cases was limited, we decided to use it as one age group. Sensitivity analysis was performed by applying mortality-related analyses on a subgroup after excluding deliveries with any neonatal congenital anomaly.

All tests were two-sided; *P*-values < 0.05 were considered statistically significant. Statistical analyses were performed using SPSS version 25.0 statistical package (Armonk, NY: IBM Corp.).

## Results

**Fig. 1:**
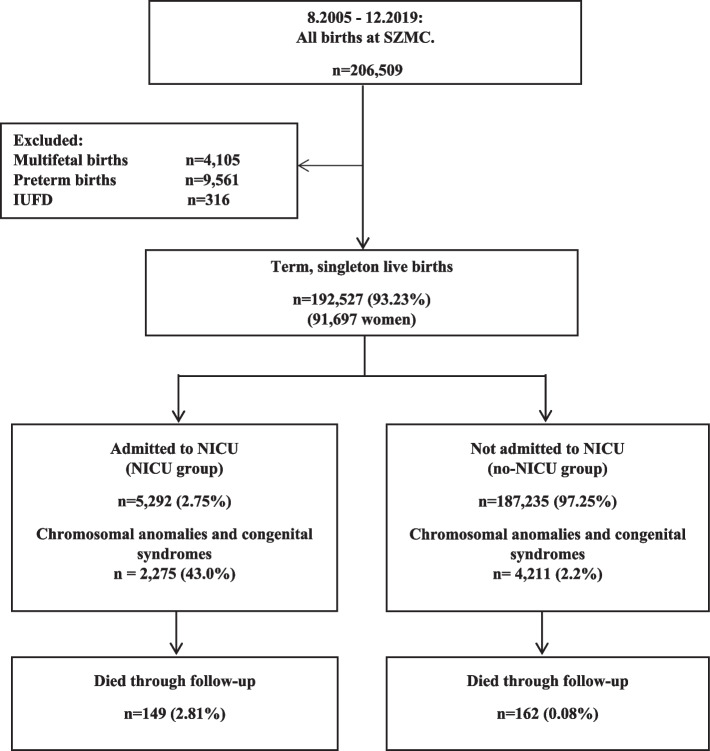
Study population flow diagram

During the study period, 206,509 births were recorded at the Shaare Zedek Medical Center. After the application of study exclusion criteria, we included 192,527 (93.23%) term singleton live births in the study (91,697 women). During a median follow-up of 7.2 years, 311 childhood deaths were recorded; 47.9% (*n* = 149) of those who died had records of a NICU admission as compared with 2.7% (*n* = 5143) of the survivors (*P* < 0.001) (Fig. 1). Detailed neonatal mortality for the newborns admitted/not admitted to NICU by follow-up time period until death can be found at Additional file 1: Table S2.

An analysis for predicting mortality among term neonates in relation to NICU admission was performed. The median [IQR] follow-up time was 7.2 [3.9–10.7] years. A detailed comparison between the characteristics of the decedents and the survivors is presented in Table 1.

**Table 1:** Demographic, obstetrical and neonatal characteristics of the deceased vs. survivors

	**Variable**	**Deceased (*n* = 311)**	**Survivors (*n* = 192,216)**	***P***
**Demographics and clinical history**	**Maternal age (Y)**	28.8 ± 6.3	28.9 ± 5.7	0.58
	**Maternal age > 35 (%)**	54 (17.4)	29,073 (15.1)	0.27
	**Jewish population group (%)**	238 (76.5)	177,185 (92.2)	< 0.0001
	**Arab population group (%)**	73 (23.5)	15,031 (7.8)	
	**Ethiopian ethnicity (%)**	5 (1.6)	2141 (1.1)	0.41
	**Education > 12 years (%)**	282 (92.5)	179,931 (97.8)	< 0.0001
	**GDM / DM (%)**	6 (1.9)	6665 (3.5)	0.13
	**Hypertensive disorder (%)**	6 (1.9)	3740 (1.9)	0.98
	**Maternal background disease (%)**	61 (19.6)	29,633 (15.4)	0.041
**Obstetrical history**	**Gestational age at birth (W)**	39.3 ± 1.5	39.5 ± 1.2	0.0028
	**Parity**	3.7 ± 2.5	3.5 ± 2.4	0.019
	**ART (%)**	5 (1.6)	5634 (2.9)	0.17
	**Prior CS (%)**	48 (15.4)	22,438 (11.7)	0.039
	**Prior miscarriage (%)**	84 (27)	56,059 (29.2)	0.40
	**High-risk hospitalization during pregnancy (%)**	331 (8.2)	7207 (4.8)	< 0.001
	**Inter-pregnancy interval (W)**	74.5 ± 50.64	85.3 ± 61.6	0.037
	**Past neonatal death (%)**	5 (1.6)	918 (0.5)	0.0040
	**Antenatal care (%)**	244 (78.5)	158,711 (82.6)	0.056
**Vital signs at admission**	**Fever (**^**o**^**C)**	36.6 ± 0.4	36.5 ± 0.4	0.18
	**Heart rate (BPM)**	88.6 ± 13.3	89.2 ± 13.8	0.61
	**Systolic > 160 (%)**	2 (0.8)	733 (0.5)	0.51
	**Diastolic > 105 (%)**	0 (0.0)	190 (0.1)	0.57
**Obstetrical data**	**Mode of delivery (%)**			< 0.0001
	**Spontaneous**	231 (74.3)	162,458 (84.5)	
	**Instrumental**	16 (5.1)	11,393 (5.9)	
	**CS**	64 (20.6)	18,365 (9.6)	
	**TOLAC (%)**	29 (9.3)	15,111 (7.9)	0.34
	**Epidural (%)**	147 (47.3)	100,668 (52.4)	0.072
	**Oxytocin during labor (%)**	96 (30.9)	45,799 (23.8)	0.0037
	**Thick meconium (%)**	20 (6.4)	9588 (5.0)	0.24
	**Head presentation (%)**	285 (92.2)	187,861 (97.9)	< 0.0001
	**Normal fetal monitor (%)**	179 (76.2)	143,039 (89.3)	< 0.0001
	**Modified Bishop > 6 (%)**	56 (40.6)	46,655 (48.5)	0.064
	**Labor duration (Min)**	301.6 ± 1376.5	275.9 ± 352.9	0.72
	**Stage II duration (Min)**	27.4 ± 41.1	33.4 ± 49.6	0.07
	**Stage III duration (Min)**	11.3 ± 10.2	10.7 ± 26.1	0.742
	**On-call hours at births (%)**	226 (72.7)	137,927 (71.8)	0.721
**Neonatal data**	**NICU admission (%)**	149 (47.9)	5143 (2.7)	< 0.001
	**Female sex (%)**	136 (43.7)	93,732 (48.8)	0.076
	**Birth weight (gr)**	3067.8 ± 614.1	3333.9 ± 429.2	< 0.001
	**5' APGAR ≤ 7 (%)**	75 (24.1)	2734 (1.4)	< 0.001
	**Macrosomia (%)**	19 (6.3)	12,154 (6.3)	0.98
	**SGA (%)**	85 (27.3)	11,983 (6.2)	< 0.001
	**Pediatrician during labor (%)**	76 (24.4)	15,570 (8.1)	< 0.001
**Neonatal adverse outcomes**	**Jaundice (%)**	16 (6.7)	8279 (4.9)	0.195
	**Infection (%)**	26 (10.9)	510 (0.3)	< 0.001
	**Hypoglycemia diabetes (%)**	15 (6.3)	8382 (5.0)	0.343
	**Respiratory (%)**	72 (30.3)	2467 (1.5)	< 0.001
	**Cardiovascular (%)**	71 (29.8)	2618 (1.6)	< 0.001
	**Skeleton skin (%)**	42 (17.6)	8682 (1.5)	< 0.001
	**Digestive (%)**	0 (0.0)	1 (0.0)	0.970
	**Other anomalies (%)**	61 (25.6)	4298 (2.5)	< 0.001
	**Chromosomal anomaly (%)**	21 (8.8)	257 (0.2)	< 0.001
	**Meconium aspiration (%)**	5 (2.1)	319 (0.2)	< 0.001
	**Brain (%)**	34 (14.3)	434 (0.3)	< 0.001
	**Electrolytes disorders (%)**	15 (6.3)	140 (0.1)	< 0.001
	**PH any (%)**	135 (43.4)	18,587 (9.7)	< 0.001

Data presented as the group means ± SD or as frequencies (percentages). *GDM/DM* Gestational/pre-gestational diabetes mellitus, *ART* Assisted reproductive technology, *CS* Cesarean section, *TOLAC* Trial of labor after cesarean, *NICU* Neonatal intensive care unit, *SGA* Small for gestational age, *PH* Potential of hydrogen, *Y* Years, *W* Weeks, *N* Number, *Min* Minutes, *C* Celsius, *BPM* Beats per minute, *Gr* Grams

A multivariable Cox proportional hazards model for the mortality risk, adjusted to delivery and neonatal characteristics in this cohort of neonates revealed that NICU admission was the most prominent risk factor associated with mortality HR = 19.72, 95% CI [14.66, 26.53] *P* < 0.001. After excluding 6486 births (3.4%) with records of congenital anomaly (chromosomal or genetic syndrome), the model retained a similar significant magnitude of association between NICU admission and mortality HR = 19.79 95% CI [11.71–33.45]; *P* < 0.001. Other significant risk factors were 5′ Apgar ≤ 7 (HR = 4.40, 95% CI [2.08, 9.30] *P* < 0.001), Arab population group (HR = 2.85, 95% CI [1.74, 4.66] *P* < 0.001) and SGA (HR = 2.20, 95% CI [1.30, 3.80], *P* < 0.001) (Table 2).

**Table 2:** Cox proportional hazards model for mortality risk, adjusted to delivery and neonatal characteristics, after excluding congenital anomalies and genetic syndromes

	**HR (95% CI)**	***P***
NICU admission	**19.79 [11.71, 33.49]**	< 0.001*
Jewish population group	**1**	
Arab population group	**2.85 [1.74, 4.66]**	< 0.001*
Nulliparity	**0.61 [0.36, 1.01]**	**0.053**
Anemia	**2.30 [1.44, 3.66]**	0.001*
5' APGAR ≤ 7	**4.40 [2.08, 9.30]**	< 0.001*
SGA	**2.20 [1.30, 3.80]**	0.005*

Significant differences are indicated by an asterisk (*). Model was adjusted to delivery and neonatal characteristics

*Abbreviations*: *HR* Hazards ratio, *CI* Confidence interval, *NICU* Neonatal intensive care unit, *SGA* Small for gestational age

**Fig. 2:**
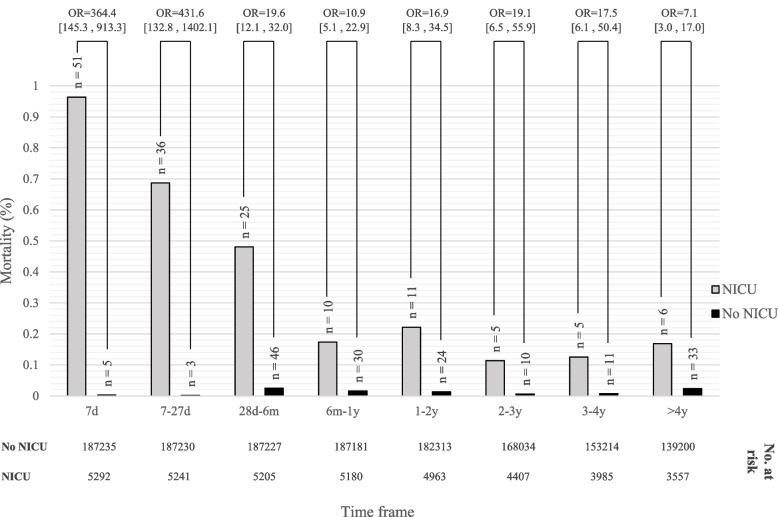
Neonatal mortality – crude rate and odds ratios for neonates admitted to the NICU versus non admitted: analysis by time elapsed from birth

**Fig. 3:**
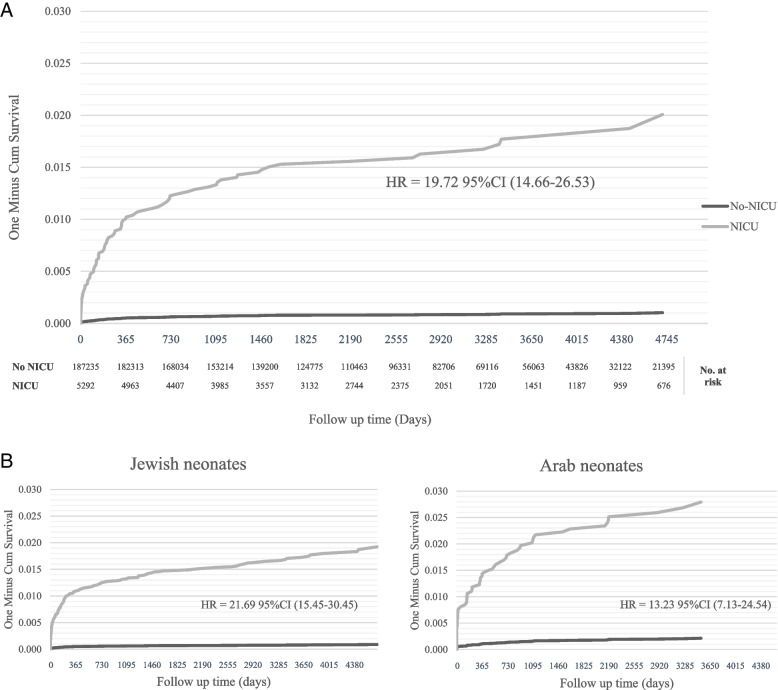
**A** Kaplan–Meier curve – cumulative incidence of neonatal and childhood mortality among neonates admitted vs. not admitted to NICU. **B** Kaplan–Meier curve – cumulative incidence of neonatal mortality among Jewish and Arab neonates admitted vs. not admitted to NICU

We further performed an analysis to compare NICU and no-NICU-admitted neonates for childhood mortality risk according to the time of death during the follow-up period. Neonatal NICU admission was significantly and consistently associated with an increased rate of mortality at any time during follow-up (Fig. 2). The neonatal NICU admission after birth was revealed to be strongly associated with neonatal mortality risk at 7 to 27 days (OR = 431.65, 95% CI [132.8, 1402.1] *P* < 0.001); the risk correlation was pronounced for mortality in the first 7 days after birth (OR = 364.39, 95% CI [145.3, 913.3] *P* < 0.001); however, remained significantly high at the later period of follow up; specifically, after the age 4 years HR, 7.1 [3.0; 17.0], *p* < 0.001. The cumulative incidence of mortality in the NICU as compared to the no-NICU admission groups is described by Kaplan–Meier curves, with significant divergence among the groups; while the no-NICU groups stabilized after 4 years of follow up, the mortality risk in the NICU admission group continued to rise through the entire follow-up period of 15 years; *p* (log-rank) < 0.001 (Fig. 3A). The separate analyses of each of the main population sub-groups showed a similar pattern of mortality risks as the general population; nevertheless, the long-term risk was significantly more evident for the Arab population as compared to the Jewish population. Notably, albeit the higher overall risk for long-term mortality; in the Arab subpopulation group this risk was less related to the NICU admission event as compared to the Jewish population (HR = 13.23 [7.13; 24.5] and HR = 21.69 [15.45; 30.45], respectively) (Fig. 3B).

**Fig. 4:**
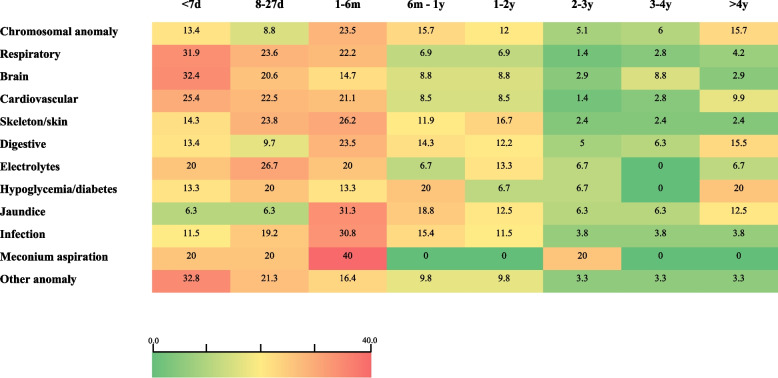
Neonatal mortality heat map by NICU admission diagnosis and time elapsed from birth

We further assessed mortality risk during follow-up in association with the diagnoses at the initial NICU admission. Notably, we found that mortality at specific periods was strongly associated with specific causes of NICU admission e.g. mortality at 0 to 7 days was mainly associated with brain and respiratory causes, while mortality at 28 days–6 months was mostly associated with meconium aspiration, infections, and jaundice (Fig. 4). In order to evaluate the significance of such diagnoses separated from other background diseases, we first used the prototype of “neonatal jaundice.” Overall for the study population, we identified 8,295 neonates with a diagnosis of “jaundice”; for 6656 (80.2%) jaundice was the only registered neonatal diagnosis, among which 92 (1.4%) were admitted to NICU. None of these neonates died during the follow-up period. Next, we evaluated the long-term mortality risk with a model that included the diagnoses grouped as disorders with a potentially high severity association strength and those with a low, potentially transient association. We found that in a model adjusted for the potential severity of the disease, the NICU admission event remained an independent and significant risk factor for long-term mortality as compared to the non-admitted neonates; HR = 8.21 95% CI [5.43; 12.4] *P* < 0.001.

## Discussion

In this study, we show that admission to NICU with any diagnosis is a significant risk indicator of long-term childhood mortality for term neonates.

The study population included 192,527 term neonates, with a 2.75% rate of NICU admission following delivery. Compared to other large-scale studies conducted [8–11], this admission rate for NICU lies within the lower range; in another study, the risk for NICU admission was 4.1%, and risk factors for admission included non-citizen status, low or no health insurance coverage, and premature rupture of membranes [12]. We suggest that the difference in NICU admission rate may be explained mainly by our study being performed at a single center, with a homogenous population covered by a national health care plan, no out-of-hospital transfers, and a highly trained neonatology service present in the delivery room. In addition, different exclusion criteria and different hospitalization protocols (e.g., induction of preterm premature rupture of membranes prior to term, at 35 weeks’ gestation) could partially explain the difference. However, due to the large population in our study, we were able to comprehensively assess pre-birth information such as maternal background, vital signs and blood tests at admission for birth, characteristics of labor, and neonatal characteristics.

As previously noted, death is considered a common occurrence among term neonates during the NICU hospitalization, with a reported incidence of 8–15% [2, 3]. The existing scoring systems such as SNAPPE-II (Score for Neonatal Acute Physiology with Perinatal Extension-II) serve as a tool to predict early mortality during the NICU hospitalization [3, 13]. Yet, to the best of our knowledge, no study has assessed the association between NICU admission for term neonates and long-term mortality also after hospital discharge. Overall, among the 0.16% of the cohort who died during the 15-year follow-up period, half had records of neonatal NICU admission, a significant and intriguing high rate as compared to neonates not admitted to the NICU; we found no other similar study to compare our rates to and consequently our study may serve as a comparison base for future research.

The Cox proportional hazards model suggested that neonates admitted to the NICU had a 20-fold increased risk for death, not only immediately subsequent to the admission but throughout the entire 15-year follow-up period. Interestingly, the association of this magnitude remained evident after adjustment for gestational age at birth and exclusion of congenital malformations, and chromosomal and genetic syndromes, which are considered well-known leading causes for NICU admission and early mortality [14, 15]. This finding reveals an additional significance for the Arab population who presented an increased risk of post-NICU-related mortality, however, less related to the NICU admission event itself as compared to the Jewish population. beyond the consanguineous marriages and low termination of pregnancy due to antenatal detected fetal abnormalities, we postulate that the Arab population’s higher mortality following NICU admission might be due to the poor post-hospital discharge follow-up and care; both because of the higher maternal rate of postpartum depression, parents’ perception of the importance of neonatal follow-up and the peripheral residence areas with low access to health care, lack of safe spaces and a higher rate of accidental deaths, especially for sick children discharged from NICU [16–20]. Nevertheless, for both populations, the risk was significantly higher for those admitted to the NICU.

Others have previously described risk factors for early mortality [3, 21], such as low Apgar score, ethnic/population groups, and SGA neonates which were significant also in the present study, at a much lower magnitude with only a two-fold increased death risk. Markedly, the cumulative mortality rate in our study continued to increase throughout the entire follow-up period for those admitted to the NICU; in contrast to a stabilization of the risk observed after 4 years of follow-up among those not admitted to the NICU. It is difficult and beyond the scope of the present study to theorize over an explanatory hypothesis. However, we may postulate that neonates admitted to NICU suggest a role for genetic and epigenetic variations for susceptibility to stress, inflammation-induced damage [22–24], and different maternal diets and supplements during pregnancy, especially for mothers with diabetes mellitus or hypertensive disorders [25–27].

While the effects of NICU admission indications and diagnoses on early mortality have been previously discussed [28], the association with late childhood mortality is less well known, especially with respect to term neonates. The most common reasons for a term infant to be admitted to the NICU after birth are temperature instability, hypoglycemia, respiratory distress, and hyperbilirubinemia [29]. Our population showed a similar pattern for diagnoses at the time of the NICU admission. Interestingly, neonates admitted to NICU with potentially early detectable and treatable diagnoses like jaundice and common metabolic complications without any background of metabolic or other diseases (dehydration and electrolytes disorders, hypoglycemia, infant to a diabetic mother) exhibited likewise a sustained long-term childhood mortality risk, at least until 4 years of age. Importantly, hyperbilirubinemia and hypoglycemia in NICU-admitted term neonates are complications that are not overlooked and carefully observed; medical management is sufficient and well tolerated [30]. However, the exact association of each diagnosis at NICU admission on childhood mortality is beyond the scope of the present study; the majority of the neonates had records of one or more diagnoses and the inherent difficulty of record study does not allow us to dissect between the primary or the secondary diagnoses. For example, diagnoses such as jaundice and electrolyte disorders may not stand as an isolated diagnosis for NICU admission. Therefore, jaundice alone has little or no association with the long-term mortality risk for the NICU admitted neonates. Furthermore, it may be obvious to the clinician intuition that the cause and the severity of the condition that determined the neonatal admission to NICU is probably the link to the long-term mortality rather than the event of the NICU admission itself. This is a question that is difficult to resolve in a study based on medical records such as ours. However, in order to evaluate the NICU admission event itself as an independent risk for long-term mortality we approached it by using models fitted for the potential severity of the recorded diagnoses. Indeed, the independent magnitude of the NICU admission risk was reduced two folds yet remained significantly high compared to the non-admitted neonates.

Altogether, we postulate that the short-term mortality increased risk for the neonates admitted to NICU may be attributed mostly to the underlying disease, the long-term mortality risk related to the NICU admission event is enthralling and further studies are required to fully explore into this pattern of long-term mortality risk and its pathogenesis.

### Strengths and limitations

This study adds to a limited number of studies which examined predictive models for NICU admission of neonates at term; diagnoses and mortality. It was designed as a large study based on validated and linked databases. The use of a single center and in-hospital-born neonates offers the advantage of uniform management care, both for obstetrics and neonatology. The antenatal, labor, and all health and hospitalization costs are covered by the National Health Insurance Plan for all mothers and neonates, as well as the health care throughout life. Due to the low immigration effect of the medical center population and the unique identification number allocated to each mother and child, we are able to cross-link databases and examine long-term follow-up for mortality. All of these properties allow us to assess the reliability of the results and reach robust conclusions.

Some limitations need to be considered. The proposition that NICU admission is associated with an increased risk of long-term mortality is difficult to study without bias from other variables. NICU admission is also a proxy for the severity of illness; however, the fact that overall NICU-admitted babies retain significantly higher rates of death through late childhood after adjustment for the severity of the diagnoses, as compared to no-NICU-admitted neonates is still remarkable; as is the magnitude of this risk. We carefully acknowledge that this association is a thought-provoking message of a single study, thus the limitations are important. Shaare Zedek Medical Center is located in Jerusalem and serves a relatively homogeneous population, consisting mainly of the Jewish population; therefore, to the projection of our results onto other populations is difficult; however, the great variety of NICU cases and diagnoses is similar to other reports and the separate analyses for sub-population groups partially mitigate this limitation. Additionally, a 15-year study period span might pose some consideration on the management protocols change; this might be partially mitigated by the fact that for each point in time, all the neonates included in the study were treated at the same center under the same protocol.

Another significant limitation of the long follow-up period of time is the fact that the outcome of mortality is "censored early". This might be partially corrected by the Cox model which accounts for the time that elapsed from birth until death or lost to follow-up. The mortality analysis was based on 311 deaths (0.16%) only. In addition, we lacked data on maternal nutrition supplements during pregnancy, BMI, and smoking status, which may have affected NICU admission. Nevertheless, our population care is covered by the National Health Insurance plan, which is characterized by more than 80% attendance for early pregnancy care and free access to prenatal nutritional supplements. Additionally, Israeli death records do not include information on the place and cause of death, consequently, incidental deaths not related to health such as motor car accidents may have obscured the data; however, this was less likely to influence the early years of life death rate.

## Conclusions

NICU admission event for term neonates, probably independent of the diagnosis at admission, is a remarkable event with a significant association with the risk for long-term childhood mortality. Neonates at term admitted to NICU may benefit from long-term focused health care and risk-reducing interventions.

## Supplementary Information

**Additional file 1: Table S1.** Candidate covariates list in cox proportional hazard models for mortality. **Table S2.** Neonatal mortality for newborns admitted/ not admitted to NICU reported by the follow-up time period.

## Data Availability

The datasets used and/or analyzed during the current study are available from the corresponding author on reasonable request.

## Abbreviations

HR: Hazard ratio
NICU: Neonatal intensive care unit
SGA: Small for gestational age
