# Supplementary material for: Neonatal intensive care admission for term neonates and subsequent childhood mortality: a retrospective linkage study
Source: BMC Med. 2023 Feb 6;21:44. doi: 10.1186/s12916-023-02744-7 (PMC9903506; doi:10.1186/s12916-023-02744-7)
Supplement: Supplementary file 1 — Additional file 1: Table S1. Candidate covariates list in cox proportional hazard models for mortality. Table S2. Neonatal mortality for newborns admitted/ not admitted to NICU reported by the follow-up time period. [file 12916_2023_2744_MOESM1_ESM.docx]

**Additional file 1:**

**Table S1:** **Candidate covariates list in cox proportional hazard models for mortality.**

| Arab ethnicity |
| --- |
| Education – 12 year and more |
| Gestational diabetes mellitus |
| IVF therapy |
| Any maternal clinical condition |
| Any pregnancy follow-up |
| nulliparity |
| Prior cesarean section |
| Gestational age 37 weeks |
| Gestational age 42/43 weeks |
| High risk hospitalization |
| Past neonatal death |
| Elective cesarean section |
| Induction of labor |
| Cesarean section |
| Anemia at admission |
| Small for gestational age |
| Head presentation |
| Female gender |
| APGAR 5 min lower/equal 7 |
| NICU admission |
| Pediatrician during labor |

**Table S2:** **Neonatal mortality for newborns admitted/ not admitted to NICU reported by the follow-up time period.**

| **Time period** | **No NICU** | **NICU** | **Mortality cases (n)** | **Mortality rate  (R_1:10,0000 births_)** | **n at risk** |
| --- | --- | --- | --- | --- | --- |
| **0 to 7 d** | 5 | 51 | 56 | 29 | 192527 |
| **7 to 28 d** | 3 | 36 | 39 | 20 | 192471 |
| **28 to 6 m** | 46 | 25 | 71 | 37 | 192432 |
| **6m o 1 y** | 30 | 10 | 40 | 21 | 192361 |
| **1y to 2 y** | 24 | 11 | 35 | 19 | 187276 |
| **2y to 3y** | 10 | 5 | 15 | 9 | 172441 |
| **3y to 4 y** | 11 | 5 | 16 | 10 | 157199 |
| **> 4 years** | 33 | 6 | 39 | 27 | 142757 |
| **Total** | 162 | 149 | 311 |  |  |

Abbreviations: NICU, Neonatal intensive care unit; d, days; m, month; y, years; n, number; R, ratio.
